# A Hospital Problem

**Published:** 1908-10-31

**Authors:** 


### A Hospital Problem.

In the introductory address to the Medical Society of the new President, Mr. C. B. Lockwood, attention was called to certain matters connected with hospital administration and work which, while familiar enough to the medical and surgical staffs at most hospitals, have hardly attracted sufficient attention at the hands of hygienists and public health specialists. On the principle, it must be supposed, that the cobbler's children are ever the worst shod, it is undeniably true that conditions of work prevail in the out-patient rooms of many hospitals which would rightly incur the intervention of the law if discovered on the premises of a private manufacturer. It is true, as Mr. Lockwood said, that the stress entailed on the junior members of the staff at many London hospitals—apart altogether from the question of teaching when a medical school is attached—is excessive, and tending constantly to increase. As evidence of these severe demands on the energy of hospital residents and junior staffs, it was pointed out that a very considerable incidence of disease occurs. The medical world hardly needs to be reminded that three members of the junior staffs at teaching hospitals in London have lately succumbed to various illnesses within a few days of each other, but it may be doubted whether the full magnitude of the evil is even now quite realised. A few years ago a census of the cases of serious illness occurring among the residents at a large hospital in London revealed statistics positively alarming: over a period of five years something like 30 or 40 per cent. of the men had developed during their term of office a serious illness of some kind; most frequently enteric fever, diphtheria, scarlet fever, or tuberculosis. Admitting that there may have been an exceptional run of infection in that particular instance, it still remains true that the morbidity among hospital staffs, resident and non-resident, is far higher than it has any business to be among a body of hitherto healthy men in the prime of life. The feeding, too, of the residents in large hospitals is, in London at least, very far from what it should be.

How to remedy this serious state of things is not very easy to indicate in a practicable way. To say that old hospitals must be pulled down and that, in constructing new ones, the health of the medical staff must be as much considered as that of the patients and nurses, is a counsel of perfection which will probably be attained in due time, but certainly not just yet. Another suggestion is that to ensure adequate rest and holiday for the junior staff they should be properly rewarded for their services: probably no medical man will disagree, but so long as young qualified men will consent to staff our hospitals for nothing, or next to nothing, hospital managers can scarcely be expected to treat them with generosity in these times of financial stringency. Increase of staff so as to relieve the strain of overwork is said to be impossible owing to lack of accommodation in most hospitals. We are glad also to note the sympathetic references to the heavy labours of the assistant-surgeons and the anæsthetists, for whom, if they are to do all their unpaid work and make a living as well, original research is rapidly becoming difficult from want of time; a state of affairs which, as was duly pointed out, is not likely to make for good in the future of British surgery.

The situation to which attention has thus been called by Mr. Lockwood might almost be called Gilbertian if it had not aspects so very far removed from amusing. To compare the instruction of students by their teachers in public health and hygiene with the conditions in the next building where every fundamental principle of those sciences is being totally disregarded, to the direct detriment of medical men's health, is a contrast so grotesque that it seems difficult to believe that it should be allowed to continue. In saying that we do not think it can continue much longer after Mr. Lockwood's address, we are, perhaps, optimistically confusing the thought and the wish; but, at any rate, nothing but good can come of the courageous manner in which the subject has been tackled, and we look forward to practical results in due course of time.

